# Case of Congenital Hernia, with a Peritoneal Sac Enveloping the Protruded Intestine, inside the Tunica Vaginalis

**Published:** 1818-10

**Authors:** William Henry Hunt

**Affiliations:** Member of the Royal College of Surgeons, London.


THE LONDON

### Medical and Physical Journal.

4 OF VOL. XL.] OCTOBER, 1818. [NO. 236.

"For many fortunate discoveries in medicine, and for the detection of nume-
"rous errors, the world is indebted to the rapid circulation of Monthly
"Journals; and there never existed any work to which the Faculty in
"EUROPE and AMERICA were under deeper obligations than to the
"Medical and Physical Journal of London, now forming a long, but an
"invaluable, series."—RUSH.

### *For the London Medical and Physical Journal.*

*Case of Congenital Hernia, with a Peritoneal Sac enveloping the protruded Intestine, inside the Tunica Vaginalis.* By WILLIAM HENRY HUNT, Esq. Member of the Royal College of Surgeons, London.

HAVING lately met with a case of the above uncommon variety,—two only of which, I believe, have been recorded, (one by Mr. Hey, the other by Mr. A. Cooper,)—I send you the details, trusting you will deem it worth inserting in your valuable Journal.

On June 2d, a male infant, only twenty-nine days old, was brought to me with a hernia occupying the right groin and upper part of the scrotum, in a state of strangulation. The account the nurse gave was, "that the swelling occurred two days ago, the cause of which was attributed to the bandage for the defence of the umbilicus having been applied too tight round the abdomen: the child instantly seemed in pain; and, vomiting taking place, with stoppage of the bowels, urged her to take him for advice to a druggist, who, thinking it to be a phlegmonous inflammation, had ordered a cold lotion, &c." The integuments covering it were red and inflamed, and an attempt to reduce it by the taxis gave considerable pain; the belly was much swollen, tense, and painful; a gulping-up of bilious matter and stercoraceous vomiting had subsisted some hours. Bleeding and the tobacco-glyster I deemed inadmissible in so young an infant: the cold application was the only thing I tried, which proving ineffectual with the assistance of the taxis, and symptoms growing every minute worse, I was of opinion, that not a moment was to be lost, and that performing the operation immediately was the *unicum sed anceps remedium*. I, therefore, solicited Mr. Swayne to see the patient, to give his opinion as to the necessity, and assist me in performing it. When the operation had been so far accomplished that I had opened the tunica vaginalis, instead of intestine protruding through the incision, we found a smooth polished serous membrane presenting, with its vessels running in a longitudinal direction,—in fact, a peritoneal sac; which being opened, and the contents freely exposed, consisted of a knuckle of the small intestines, about three inches in length, of a dark chocolate colour: we each of us now tried to return it into the abdomen without dividing the ring, which proving unsuccessful, and deeming it of importance not to cut the ring more than was absolutely requisite to allow of replacing the gut, I only divided a few fibres of it, (which was accomplished with some difficulty, as the tendon embraced the intestine very closely.) Attempts were again made to reduce it, which not being successful, I was obliged to divide the ring to a greater extent, when, with some perseverance, the intestine was reduced. The testis in this case was plainly to be felt below the lower end of the hernia, prior to performing the operation, which puzzled me,—for Lawrence says, "Congenital may be distinguished from scrotal hernia by the impossibility of feeling the testicle, which part can be clearly discerned in common cases; its existence in infancy affords also a strong suspicion of its being of this kind. And we have great reason to conclude, that a scrotal hernia in a child is congenital."—3d. edition, page 474-5.

Yet he gives a case to show, that the rule for scrotal hernia in children being congenital does not hold good invariably; which induced me, prior to the operation, to think this was a case of the common kind, outside of the tunica vaginalis: when, however, the operation had been performed, the cure was evident; the testis and cord were laid bare. The stercoraceous vomiting which had supervened some hours before the operation, continued for twelve hours after it, when it ceased, and the infant then had stools.

The detail of the daily symptoms and treatment until the patient recovered, I fear, would trespass too much upon your pages; suffice it, therefore, to say, that the bowels were kept freely open by ol. ricini; the tension, pain, and swelling of the abdomen gradually subsided, and the wound was healed in about a fortnight.

*Bristol; August* 10, 1818.

