# Errata

**Published:** 1818-12

**Authors:** 


					266, ? .32, tor the curcwas evident, read the case, &c.

				

